# Molecular prevalence, phylogeny and hematological impact of *Toxoplasma gondii* and *Plasmodium* spp. in common quails from Punjab, Pakistan

**DOI:** 10.1371/journal.pone.0304179

**Published:** 2024-05-31

**Authors:** Ghafoor Ahmad, Ardas Masud, Muhammad Naeem, Abdul Ghafar, Hira Muqaddas, Muhammad Fiaz Qamar, Ayman A. Swelum, Maged A. Al-Garadi, Majid S. Jabir, Mourad Ben Said, Adil Khan, Furhan Iqbal

**Affiliations:** 1 Institute of Zoology, Bahauddin Zakariya University, Multan, Pakistan; 2 Department of Zoology, Islamia University Bahawalpur, Bahawalpur, Pakistan; 3 Department of Zoology, The Women University Multan, Multan, Pakistan; 4 College of Veterinary and Animal Sciences, Jhang, Punjab, Pakistan; 5 Department of Animal Production, College of Food and Agriculture Sciences, King Saud University, Riyadh, Saudi Arabia; 6 Applied Science Department, University of Technology-Iraq, Baghdad, Iraq; 7 Laboratory of Microbiology, National School of Veterinary Medicine of Sidi Thabet, University of Manouba, Manouba, Tunisia; 8 Department of Basic Sciences, Higher Institute of Biotechnology of Sidi Thabet, University of Manouba, Manouba, Tunisia; 9 Department of Botany and Zoology, Bacha Khan University, Charsadda, Khyber Pakhtunkhwa, Pakistan; 10 Department of Biology, Mount Allison University Sackville, New Brunswick, Canada; Kerman University of Medical Sciences, ISLAMIC REPUBLIC OF IRAN

## Abstract

This study investigates the molecular prevalence and phylogenetic characteristics of two prominent blood-borne pathogens, *Toxoplasma gondii* (*T*. *gondii*) and *Plasmodium* spp., in common quails (*Coturnix coturnix*) sampled from both wild (N = 236) and farmed (N = 197) populations across four districts (Layyah, Dera Ghazi Khan, Lahore, and Multan) in Punjab, Pakistan, during the hunting seasons from 2021 to 2023. Additionally, the impact of these pathogens on the complete blood count (CBC) of the hosts is examined. Out of 433 quails tested, 25 (5.8%) exhibited amplification of the internal transcribed spacer (*ITS-1*) gene for *T*. *gondii*, while 15 (3.5%) showed amplification of the *Cytochrome b* gene for *Plasmodium* spp. A risk factor analysis indicated that the prevalence of both pathogens was not confined to specific sampling sites or bird sexes (P > 0.05). District-wise analysis highlighted that hens were more susceptible to both *T*. *gondii* and *Plasmodium* spp. infections than cocks. Wild quails exhibited a higher susceptibility to *T*. *gondii* compared to farmed birds. Significant CBC variations were recorded in infected birds as compared to uninfected ones. BLAST analysis of generated sequences has confirmed the identity of recovered PCR amplicons as *T*. *gondii* and *Plasmodium relictum*. Phylogenetic analysis revealed that Pakistani isolates clustered with those reported from various countries globally. This study provides the first documentation of *T*. *gondii* and *Plasmodium* sp. infections in Pakistani quails, underscoring the need for detailed investigations across different regions to enhance our understanding of infection rates and the zoonotic potential of these parasites.

## 1. Introduction

The Quail (*Coturnix coturnix*) is a partially migratory bird species belonging to the Galliformes Order and the Phasianidae Family. It is a medium-sized, visually appealing ground-nesting game bird, known locally as ’Batair’ in Urdu [1]. Quails are highly mobile birds found across Eurasia and Africa. They possess unique taste characteristics in their meat and eggs, exhibit rapid reproduction, and offer short-term capital recovery, making quail farming a popular choice for farmers in some countries [2, 3]. In Pakistan, the commercial poultry production started for chicken in the 1960’s and later on extended to quails, ducks, turkeys and geese and since then this sector is providing a significant portion of daily proteins to the Pakistani population [4]. The rearing conditions varies from farm to farm as well as economic conditions of the area as well as the farmer. Usually 5–7 quails are kept in a cage and they are fed by crumbles or mesh and kept them dry and secure from cats, dogs and rodents that very common in Pakistan [5]. Despite the rapid growth in quail egg and meat production, there is still a lack of well-established quail farming practices and welfare standards. The biggest hindrance to the development quail farming industry includes the lack of vector and parasite control programs as well as the existence of unreliable quail market and poor market accessibility of quail and its products [4].

In the Asian subcontinent, including Pakistan and India, common quails have been both domestically raised and naturally present in the wild for many years [1]. Birds, serving as hosts for a diverse array of parasites, play a crucial role in the onward transmission of these parasites within wildlife and potentially to humans [6]. Various factors, including the stability of the infectious agent, population density, animal handling procedures, virulence, and route of exposure, influence the likelihood of zoonotic disease transmission [7]. The quail industry has reported significant economic losses due to various endo-parasites, including bacteria, protozoa, and helminths [8]. Among protozoan diseases, toxoplasmosis is particularly important as it is zoonotic and caused by the intra-cellular obligate apicomplexan parasite *T*. *gondii* [5]. Due to their ground feeding habits, birds, including those consumed by humans, can be infected with *T*. *gondii* and transmit the infection if their meat is not properly cooked [9, 10]. Additionally, over 250 species of haemosporidians parasites, including *Plasmodium*, *Haemoproteus*, *Leucocytozoon*, and *Fallisia*, have been reported in birds [11]. Avian malarial parasites, specifically avian *Plasmodium*, are capable of developing and completing their life cycles in various bird species and their invertebrate vectors [12, 13]. Mosquitoes from the Culicidae family, including genera such as *Culex*, *Coquillettidia*, *Aedes*, *Mansonia*, *Culisetta*, *Anopheles*, and *Psorophora*, are involved in the transmission of these parasites [14]. Studies have indicated that preventing vectors and blood parasites significantly enhances the survival rates of birds, particularly during the nestling phase and early life stages [15]. Therefore, monitoring parasitic infections in birds serves as an early warning for environmental risks [16].

The advent of molecular tools has revolutionized the detection and differentiation of parasitic species, enabling researchers to effectively study them [17]. Despite recent advancements in research on *T*. *gondii* and *Plasmodium* spp. in birds, our understanding of their diversity and parasite-host interactions remains limited, particularly in Pakistan. It has already been an established fact that a number of *Plasmodium* species infecting primates have been found to be responsible for human infection [18]. The zoonotic potential of *T*. *gondii* has already been proven as it has infected one-third of the total world population. Birds can get this infection mainly by ingestion of food or water contaminated with oocysts [10]. While a few studies have investigated the prevalence of *T*. *gondii* in Pakistan [19], there is a significant lack of information regarding *Plasmodium* spp. in Pakistani birds. Quails from Pakistan have never been investigated before for the presence of both *T*. *gondii* and *Plasmodium* spp. Therefore, this study aims to fill this knowledge gap by investigating the prevalence of *T*. *gondii* and *Plasmodium* spp. and establishing their phylogenetic relationships using PCR techniques.

## 2. Materials and methods

### 2.1. Study areas and subjects

**Fig 1:**
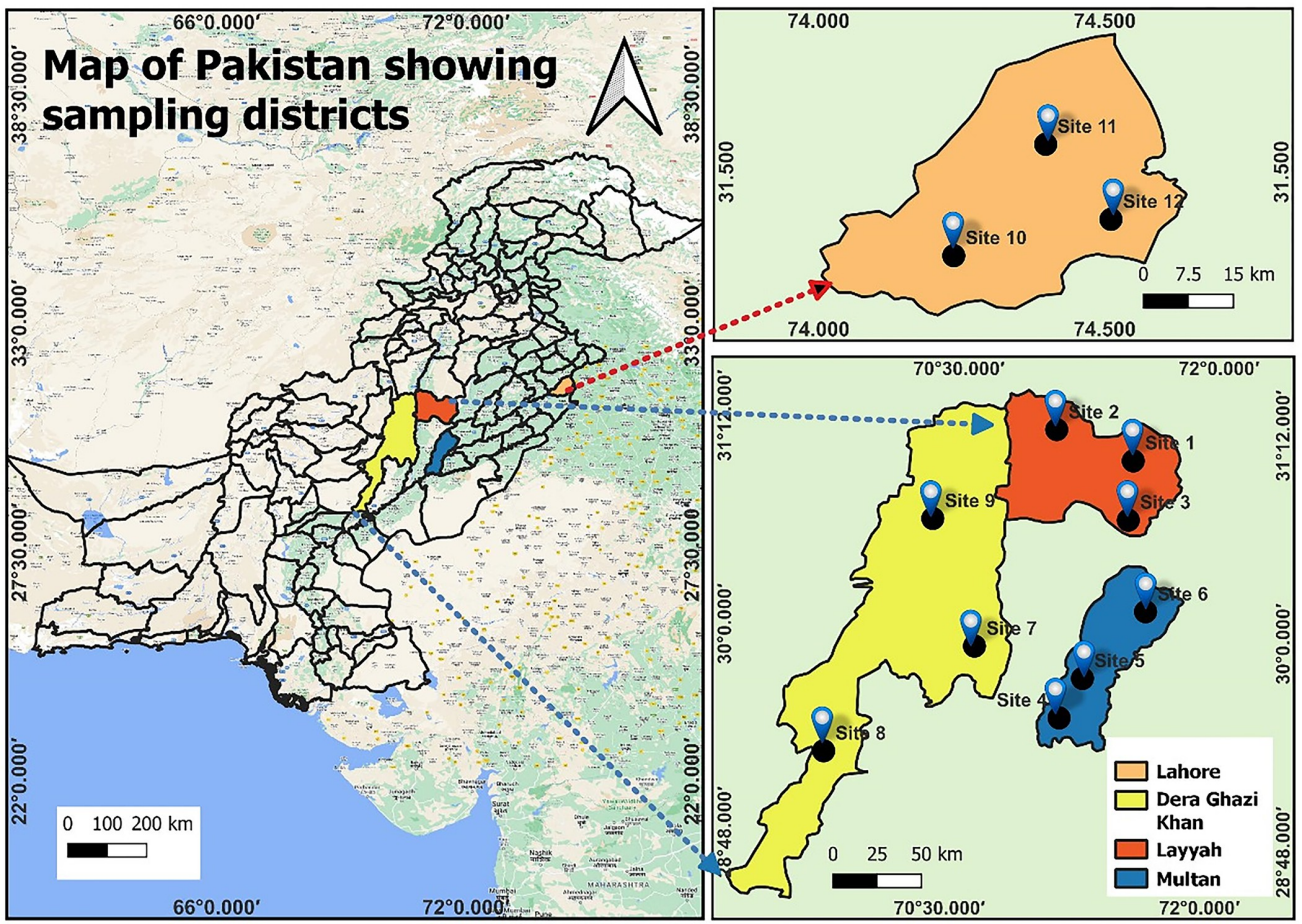
Map of Pakistan with highlighted sampling districts. Magnified map is showing the districts and sites from where the common Quail samples were collected during present study.

A total of 433 common quails, including both wild and farmed birds, were randomly selected from four districts in Punjab: Layyah, Dera Ghazi Khan, Lahore, and Multan (Fig 1). The birds were collected during the hunting seasons, which spanned from August to October, for three consecutive years (2021, 2022, and 2023). The Andrew Fisher’s formula was used to estimate the sample size and the estimated number of birds was 385. The wild quails (N = 236) were captured using mist nets, while the farmed birds (N = 197) were obtained from bird markets located in each of the sampling districts. All the birds included in this study appeared healthy (S1 Fig).

### 2.2. Data and blood collection

The experimental procedures and protocols applied in this study were approved by the Ethical Research Committee of the Institute of Zoology at Bahauddin Zakariya University Multan (Pakistan), as indicated by the approval letter with the reference number Zool./Ethics/20-11. At each sampling site, a questionnaire was filled out to collect basic information about each captured bird, including details of the sampling site, bird sex, body weight, and the presence of ectoparasites. Blood samples were collected in labeled EDTA tubes for subsequent analysis, including complete blood count analysis and DNA extraction. Blood was collected from live birds mostly from brachial vein and occasionally from wings or jugular. Blood collection site(s) was cleaned and sterilized and sterile syringe needle was used to puncture the vein (22 to 26 gauge). The birds were not sacrificed and released after blood collection.

### 2.3. Complete blood count analysis

Hematological parameters of the captured birds were analyzed using an automated hematological analyzer (MythicTM 18 Vet, Orphee, Switzerland). The following parameters were recorded for each bird during the study: red blood cell count, white blood cell count, lymphocyte percentage, monocyte percentage, hemoglobin level, platelet count, mean corpuscular volume, mean corpuscular hemoglobin, and mean corpuscular hemoglobin concentration.

### 2.4. DNA extraction from blood and molecular parasites’ detection

Genomic DNA was extracted from the blood samples of each bird using the previously described protocol by Úngari et al. [20]. The extracted DNA samples were confirmed through agarose gel electrophoresis and then subjected to analysis for the presence of *T*. *gondii* (targeting the *ITS-1* gene) and *Plasmodium* spp. (targeting the *Cytochrome b* gene) using species-specific and genus-specific primers respectively and following the protocols reported in previous studies [21, 22] (S1 Table). DNA amplification was carried out in a DNA thermal cycler (Gene Amp® PCR system 2700 Applied Biosystems Inc., UK). For the molecular detection of *T*. *gondii*, a reaction mixture of 25μl was prepared containing 13 mM Tris–HCl (pH8.3), 65 mM KCl, 2.5 mM MgCl_2_, 300 μM of each dNTP, 1U of Thermo-scientific DNA Polymerase (Nanjing Vazyme Biotech Co., Ltd., China), 0.5 μM of *ITS-1* gene primer and 5 μl of template DNA. Reaction conditions comprised of initial denaturation step at 94°C for 3 min followed by 30 cycles of denaturation at 94°C for 30 sec, primer annealing at 55°C for 45 sec and extension at 72°C for 30 sec. A final extension at 72°C for 7 min was performed following Halov´a et al. [21].

For the molecular detection of *Plasmodium* sp., a reaction mixture of 25μl was prepared containing 1X reaction buffer, 2.5 mM MgCl_2_, 300 μM of each dNTP, 1U of Thermo-scientific DNA Polymerase (Nanjing Vazyme Biotech Co., Ltd., China), 0.5 μM of ITS-1 gene primer and 5 μl of template DNA. The PCR conditions were comprised of initial denaturation step of 95°C for 15 min (for activation of Thermo-scientific Taq DNA Polymerase). This is followed by 35 cycles of denaturation at 94°C for 30 s, annealing at 59°C for 90 s, and extension at 72°C for 30 s. A final extension was performed at 72°C for 10 min [22]. For each reaction, distilled water served as a negative control, while DNA from *T*. *gondii* positive birds (Accession numbers OR761965.1, OR761966.1, available in our laboratory from previous studies) and *Plasmodium* spp. from birds that was kindly provided by Dr. Alireza Sazmand from the faculty of Veterinary Science, Bu-Ali Sina University Hamedan, Iran, was used as a positive controls respectively.

### 2.5. DNA sequencing and phylogenetic analysis

PCR amplicons that tested positive for *T*. *gondii* and *Plasmodium* sp. were sent to a commercial company, First Base, located in Malaysia, for sequencing. The obtained chromatograms were analyzed using Chromas Lite v2.01 (http://www.technelysium.com.au/chromas_lite.html). To ensure maximum data accuracy, sequencing was performed on both the forward and reverse strands of each amplicon. The complementary strands of the sequenced products were manually assembled using DNAMAN software (Version 5.2.2; Lynnon Biosoft, Que., Canada). The primer region sequences were automatically removed, and the overlapping parts were selected.

To identify previously reported sequences that matched those obtained in the present study, a BLAST analysis was conducted using the GenBank database (http://blast.ncbi.nlm.nih.gov/) [23]. Multiple sequence alignment of the amplicons was performed using the DNAMAN program. A phylogenetic tree was constructed using the DNAMAN program, employing the distance method with the Maximum-likelihood (ML) algorithm [24, 25]. Bootstrap analysis with 1000 iterations was conducted to assess the statistical support for the tree topology [26].

### 2.6. Statistical analysis

The statistical analysis of the data was conducted using the Minitab software package (Minitab, Pennsylvania, USA). The data were expressed either as mean values ± standard error of the mean (SEM) or % ± C.I. as applicable. A probability level of P ≤ 0.05 was considered significant. To compare the PCR-based pathogen prevalence among different sampling districts, a One-Way ANOVA (Analysis of Variance) was performed. The association between the presence of each pathogen and the studied epidemiological factors was assessed using contingency table analysis, specifically the Fisher’s exact test for 2 × 2 tables. To compare hematological parameters between pathogen-positive and pathogen-negative animals, a two-sample t-test was utilized.

## 3. Results

### 3.1. Prevalence of *Toxoplasma gondii* in common quails

During the present study, a specific 300 base pairs amplicon of the *ITS-1* gene of *T*. *gondii* was amplified using polymerase chain reaction (PCR) in 25 out of 433 (5.8%) blood samples collected from common quails in four districts of Punjab. Among the sampling sites, Layyah district had the highest *T*. *gondii* infection rate (9%), followed by Dera Ghazi Khan (8%), Lahore (6%), and Multan (2%). The results of a One-Way ANOVA indicated that the prevalence of *T*. *gondii* was not restricted to a particular sampling site (P = 0.112) (Table 1).

**Table 1:** *Toxoplasma gondii* and *Plasmodium* spp. over all prevalence rates in common quails enrolled from four districts of Punjab, Pakistan.

Sampling districts	Total number	*T*. *gondii*		P-value	*Plasmodium* spp.		P-value
		Positive	Rate (%±C.I.^1^)		Positive	Rate (%±C.I.^1^)	
Layyah	67	6	8.96±0.006	0.112	2	2.99±0.041	0.639
Dera Ghazi Khan	137	11	8.03±0.045		3	2.19±0.025	
Lahore	88	5	5.68±0.049		3	3.41±0.037	
Multan	141	3	2.13±0.023		7	4.96±0.035	
Total	433	25	5.77±0.021		15	3.50±0.017	

Abbreviations

^1^: C.I.: 95% confidence interval.

### 3.2. Risk factor analysis associated with *Toxoplasma gondii* infection

By analyzing the results of Fisher’s exact test, it was found that there was over all a significant difference (P = 0.05) in the prevalence of *T*. *gondii* between wild and farmed quails. Wild quails (7.62%) were more prone to parasitic infection compared to farmed birds (3.6%). This trend was specifically observed in quails from Multan district (P = 0.05). However, for the other three districts, the prevalence of this parasite did not vary significantly (P > 0.05) between wild and farmed quails (Table 2). Furthermore, the overall data analysis revealed that the prevalence of *T*. *gondii* in both wild (P = 0.105) and farmed quails (P = 0.205) was not limited to a particular bird sex. When analyzing the data district-wise, it was observed that wild hens from Multan were more prone to infection than wild cocks (P = 0.05). For the remaining districts, *T*. *gondii* prevalence was not limited to a particular bird sex in both wild and farmed quails (P > 0.05 for each district) (Table 3).

**Table 2:** Prevalence rates of *Toxoplasma gondii* in wild and farmed common quails in overall and according to the four studied districts of Punjab, Pakistan.

Sampling area	Birds	Total	*T*. *gondii*		P-value	*Plasmodium* spp.		P-value
			Positive	Rate (%±C.I.^1^)		Positive	Rate (%±C.I.^1^)	
Layyah	Wild	58	6	10.34±0.078	0.315	0	0	P < 0.001 ***
	Farmed	9	0	0		2	22.22±0.272	
Dera Ghazi Khan	Wild	81	5	6.17±0.052	0.338	2	2.47±0.033	0.788
	Farmed	56	6	10.71±0.008		1	1.79±0.035	
Lahore	Wild	39	4	10.26±0.096	0.100	1	2.56±0.049	0.698
	Farmed	49	1	2.04±0.039		2	4.08±0.054	
Multan	Wild	58	3	5.17±0.056	0.036 *	7	12.07±0.084	P < 0.001 ***
	Farmed	83	0	0		0	0	
Total	Wild	236	18	7.63±0.033	0.05 *	10	4.24±0.025	0.336
	Farmed	197	7	3.55±0.025		5	2.54±0.021	

Abbreviations

^1^: C.I.: 95% confidence interval

*: Least statistically significant, P < 0.05

**: Statistically significant, P < 0.01

***: Highly statistically significant, P < 0.001.

**Table 3:** Prevalence rates of *Toxoplasma gondii* in wild and farmed common quails according to the sex in overall and in the four studied districts of Punjab, Pakistan.

Sampling area	Bird sex	Wild quail number	*T*. *gondii* in wild quails		P-value	Farmed quail number	*T*. *gondii* in farmed quails		P-value
			Positive	Rate (%±C.I.^1^)			Positive	Rate (%±C.I.^1^)	
Layyah	Male	29	2	6.90±0.025	0.392	5	0	0	-
	Female	29	4	13.80±0.035		4	0	0	
Dera Ghazi Khan	Male	40	3	7.5±0.019	0.626	52	6	11.54±0.017	0.476
	Female	41	2	4.88±0.015		4	0	0	
Lahore	Male	22	1	4.55±0.027	0.186	26	0	0	0.287
	Female	17	3	17.65±0.068		23	1	4.35±0.025	
Multan	Male	31	0	0	0.05 *	41	0	0	-
	Female	27	3	11.11±0.033		42	0	0	
Total	Male	122	6	4.92±0.005	0.105	124	6	4.84±0.005	0.205
	Female	114	12	10.53±0.007		73	1	1.37±0.003	

Abbreviations

^1^: C.I.: 95% confidence interval.

### 3.3. Complete blood count analysis associated with *Toxoplasma gondii* infection

The analysis of complete blood count parameters showed that *T*. *gondii*-positive wild common quails had increased white blood cells (P = 0.009), lymphocytes cells (P = 0.04), monocytes (P = 0.05), mean cell volume (P = 0.05), decreased red cell distribution width with standard deviation (P = 0.05), and decreased platelet count (P = 0.002) compared to parasite-negative wild quails. In farmed common quails, the analysis revealed that only red cell distribution width with standard deviation (P = 0.04) was significantly reduced in *T*. *gondii*-infected birds compared to uninfected birds (Table 5).

### 3.4. Genetic diversity of *Toxoplasma gondii* in common quails

**Fig 2:**
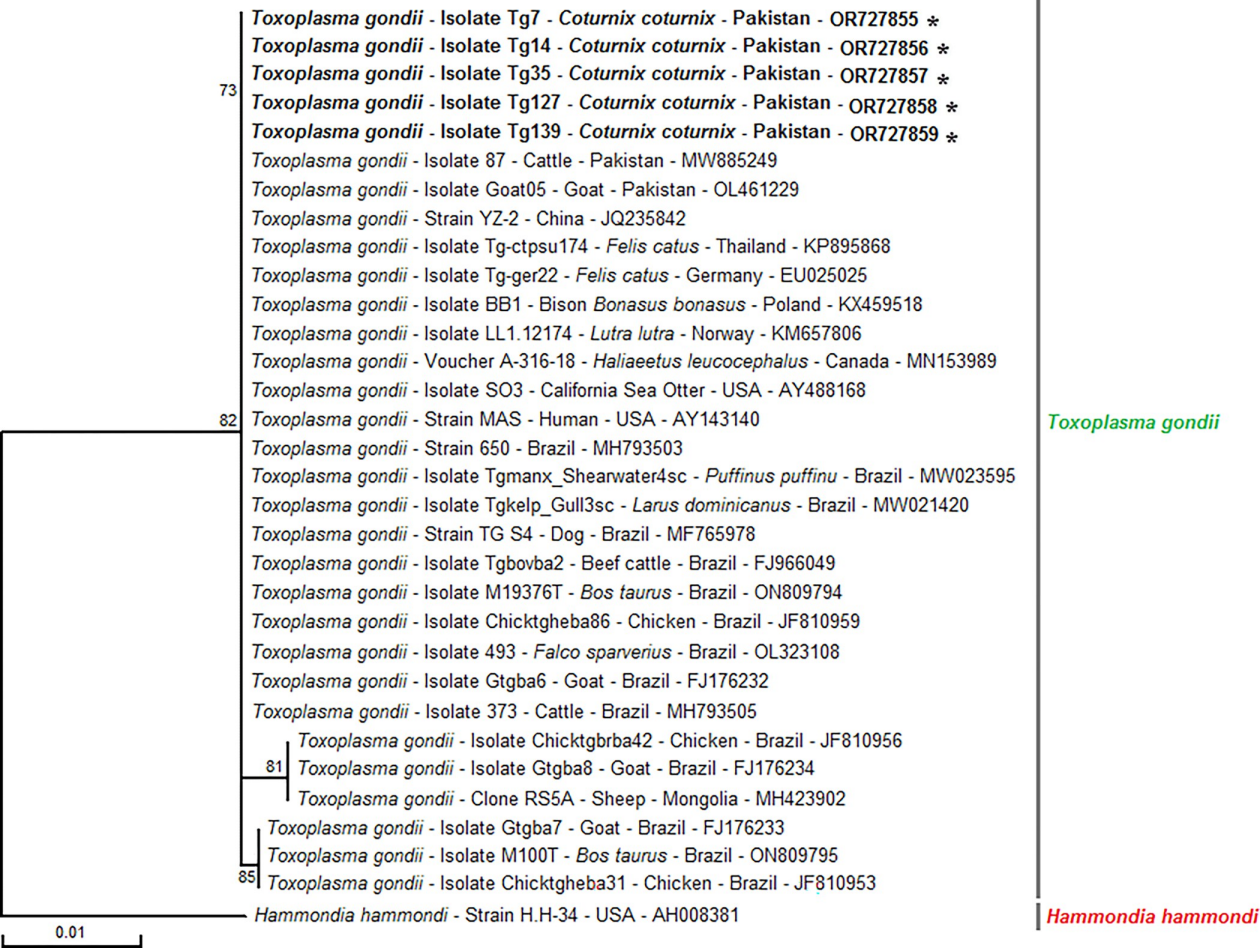
Representative maximum likelihood tree based on multiple sequence alignment of the partial ITS-1 nucleotide sequences (266 bp) of Pakistani *Toxoplasma gondii* isolates infecting common quails (*Coturnix coturnix*) with those isolated from wild and domestic animals from several worldwide countries published in GenBank. Numbers in nodes represent the percentage of 1,000 bootstrap iterations supporting the nodes (only percentages greater than 70% are shown). The host, the strain, isolate, voucher or clone identification, country of origin, and GenBank accession number are indicated in the tree for each sequence. Sequences newly obtained in this study are highlighted in bold and marked with asterisks. One *Hammondia hammondi* ITS-1 partial sequences was added as an out-group.

In the phylogenetic analysis of *T*. *gondii* based on partial sequences of the nuclear *ITS1* gene, the haplotypes OR727855, OR727856, OR727857, OR727858, and OR727859 obtained from the present study in Pakistan clustered together with previously reported haplotypes from various countries. These countries include Pakistan (MW885249 and OL461229), China (JQ235842), Thailand (KP895868), Germany (EU025025), Poland (KX459518), Norway (KM657806), Canada (MN153989), USA (AY488168 and AY143140), Brazil (MH793503, MW021420, MF765978, FJ966049, ON809794, ON809795, JF810959, OL323108, FJ176232, MH793505, JF810953, JF810956, FJ176233, FJ176234, and MW023595), and Mongolia (MH423902). During the phylogenetic analysis, the partial sequence of the ITS1 gene from *Hammondia hammondi* strain H.H-34 (AH008381) was used as an out-group (Fig 2).

### 3.5. Prevalence of *Plasmodium* spp. in common quails

In total, 15 out of 433 (3.5%) common quail blood samples were found to be positive for *Plasmodium* spp. When comparing the prevalence of *Plasmodium* spp. among the sampling sites, it was observed that Multan district had the highest infection rate (5%), followed by Layyah (3%), Lahore (3%), and Dera Ghazi Khan (2%). However, the results of a One-Way ANOVA analysis indicated that the prevalence of this parasite was not significantly different across the sampling sites (P = 0.639) (Table 2).

### 3.6. Risk factor analysis associated to *Plasmodium* spp. infection

The data analysis revealed that wild quails had a higher prevalence of *Plasmodium* spp. infection (4.2%) compared to farmed quails (2.5%). However, the results of Fisher’s Exact test indicated that this difference in parasite prevalence did not reach statistical significance (P = 0.4). When the data was analyzed for individual districts, it was observed that farmed quails from Layyah (P = 0.001) and wild quails from Multan (P = 0.001) were more prone to *Plasmodium* spp. infection. For Dera Ghazi Khan (P = 0.788) and Lahore (P = 0.698) districts, the prevalence of this parasite did not vary significantly between wild and farmed quails (Table 2). Furthermore, the overall data analysis showed that the prevalence of *Plasmodium* spp. in both wild (P = 0.067) and farmed quails (P = 0.890) was not restricted to a particular bird sex (P > 0.05) (Table 3). However, when the data was analyzed district-wise, it was observed that farmed hens from Dera Ghazi Khan were more prone to infection than cocks (P < 0.001). In the remaining districts, the prevalence of *Plasmodium* spp. was not limited to a particular bird sex in both wild and farmed quails (Table 4).

**Table 4:** Sex-specific prevalence rates of *Plasmodium* spp. in wild and farmed common quails according in overall and according to the four studied districts of Punjab, Pakistan.

Sampling area	Bird sex	Wild quail number	*Plasmodium* spp. in wild quails		P-value	Farmed quail number	*Plasmodium* spp. in farmed quails		P-value
			Positive	Rate (%±C.I.^1^)			Positive	Rate (%±C.I.^1^)	
Layyah	Male	29	0	0	-	5	2	40±0.350	0.176
	Female	29	0	0		4	0	0	
Dera Ghazi Khan	Male	40	2	5±0.015	0.149	52	0	0	P < 0.000***
	Female	41	0	0		4	1	25±0.346	
Lahore	Male	22	1	4.54±0.027	0.379	26	1	3.85±0.021	0.930
	Female	17	0	0		23	1	4.35±0.025	
Multan	Male	31	5	16.13±0.035	0.313	41	0	0	-
	Female	27	2	7.41±0.027		42	0	0	
Total	Male	122	8	6.56±0.005	0.067	124	3	2.42±0.003	0.890
	Female	114	2	1.75±0.003		73	2	2.74±0.005	

Abbreviations

^1^: C.I.: 95% confidence interval

***: Highly statistically significant, P < 0.001.

### 3.7. Complete blood count analysis associated with *Plasmodium* spp. infection

An overall analysis of complete blood count parameters revealed significant elevations in white blood cell count (P < 0.001), lymphocytes (P < 0.001), monocyte count (P = 0.006), and mean cell volume (P < 0.001). Simultaneously, there were significant decreases in red blood cell count (P = 0.05), hemoglobin concentration (P = 0.05), and platelet count (P = 0.005) in *Plasmodium* spp. infected wild quails when compared with uninfected birds (Table 6). Data analysis further demonstrated that *Plasmodium* spp. infected farmed common quails exhibited significantly elevated mean platelet volume (P < 0.001), along with increased mean cell hemoglobin (P = 0.05) and red cell distribution width (P = 0.05) compared to uninfected birds. All other parameters showed non-significant variations (P > 0.05) when comparing parasite-positive and negative birds enrolled during the present investigation (Table 6).

### 3.8. Genetic diversity of *Plasmodium* spp. in common quails

**Fig 3:**
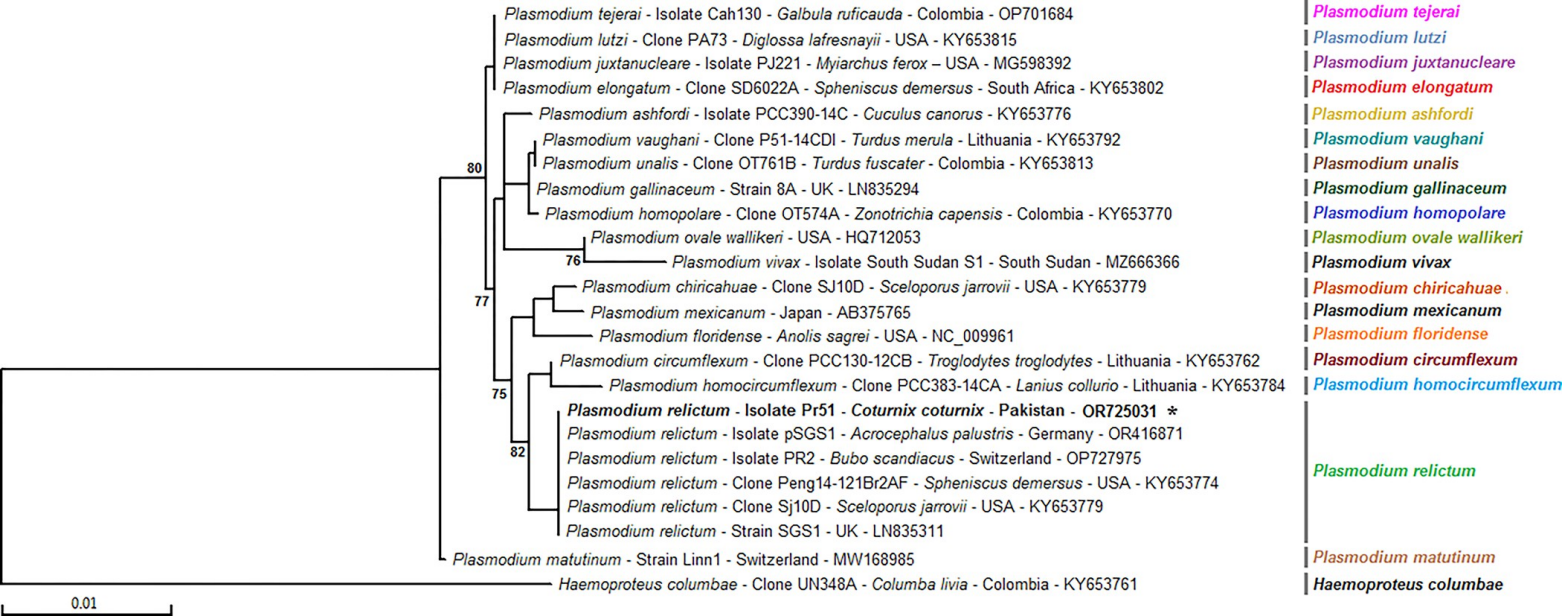
Phylogenetic tree of *Plasmodium* spp. inferred with marker genomic sequences (317 bp) using the Maximum likelihood method showing the position of revealed sequence isolated from *Plasmodium relictum* infecting a common quail (*Coturnix coturnix*) in Pakistan. Isolate from this study represented in bold and marked with an asterisk. Numbers associated with nodes represent the percentage of 1,000 bootstrap iterations supporting the nodes (only percentages greater than 70% were represented). The host, the strain, isolate or clone name, the country of origin and the GenBank accession number are indicated. One *Haemoproteus columbae* marker genomic sequence was added as an out-group.

The analysis of the partial sequence of the *Cytochrome b* gene of *Plasmodium* sp. unveiled that the current haplotype, *Plasmodium relictum* (OR725031, Pakistan), formed a cluster with several species within the genus *Plasmodium*, as reported from various locations including Germany (OR416871), Switzerland (OP727975), USA (NC009961, KY653774, and KY653779), UK (LN835311), Lithuania (KY653784 and KY653762), and Japan (AB375765). The phylogenetic tree for *Plasmodium relictum*, illustrated in Fig 3, utilized *Haemoproteus columbae* Clone UN348A (KY653761) as an out-group.

### 3.9. Co-infection of *Toxoplasma gondii* and *Plasmodium* spp. in common quails

The analysis of data from this study showed that none of the common quails, whether wild or farmed, captured from four districts in Punjab, exhibited co-infection with both *T*. *gondii* and *Plasmodium* spp.

## 4. Discussion

The current study aimed to contribute valuable insights into the prevalence and potential risks associated with *T*. *gondii* and *Plasmodium* spp. Infections in common quail populations and to bridge the gap between wildlife, domestication and public health concerns. This was conducted via thorough screening of both wild and farmed common quails, sourced from four districts in Punjab, Pakistan, to ascertain the presence of these parasites.

Despite the rich avian fauna in Pakistan, there is a notable scarcity of literature on parasite screening in local birds, particularly wild birds [3]. This dearth of research is also evident in the case of common quails, with only a handful of studies in Pakistan reporting the seroprevalence of *T*. *gondii* in these birds. To the best of our knowledge, this report marks the first instance in Pakistan where PCR has been employed for the detection of *T*. *gondii* in both wild and farmed common quails.

The oocysts of *T*. *gondii* have been identified in various environmental sources, including water, soil, vegetables, fruits, milk products, and raw meat, posing a potential risk to a diverse range of consumers [27]. Given the common presence of cats in both urban and rural areas of Punjab, these animals play an established role in the spread of *T*. *gondii*. Furthermore, the ground-feeding behavior of common quails may heighten their susceptibility to oral infection through oocyst-contaminated soil. This underscores the importance of employing advanced diagnostic techniques, such as PCR, to enhance our understanding of the prevalence and transmission dynamics of *T*. *gondii* in common quail populations, with implications for both animal and public health.

During the current investigation, we observed an overall prevalence of 5.8% for *T*. *gondii* in common quails (Table 1). The infection rate was significantly higher in wild birds (7.6%) compared to farmed birds (3.6%) (P < 0.05) (Table 2). In a prior study from Pakistan, Ibrahim et al. [27] reported a 4% infection rate in quails captured from Lahore district in Punjab. While Ibrahim et al. [27] focused on seroprevalence, our study, employing PCR, revealed a similar *T*. *gondii* infection rate of 6% in quails from Lahore district (Table 1). Both studies confirm the presence of this parasite in birds intended for human consumption, highlighting a potential zoonotic transmission of *T*. *gondii* to humans in Pakistan.

In contrast, Naveed et al. [28] did not find *T*. *gondii* infection in common quails captured from Kasur district in Punjab. The limited studies from Pakistan underscore the need for more comprehensive investigations from different regions to obtain an accurate understanding of *T*. *gondii* prevalence in quails across the country.

Globally, *T*. *gondii* has been reported in quails, both in blood and tissues, with infection rates ranging between 6% and 24%. Lamy and Kawan [29] reported a 24% prevalence of *T*. *gondii* in various organs of quails from Baghdad city in Iraq. In a related study, Lamy and Kawan [30] found a 19.33% sero-positivity for *T*. *gondii* in screened quails from the same area. Hassan et al. [31] detected *T*. *gondii* in the brains of 7% of quails from Egypt. Cong et al. [6] reported a 6.4% infection rate in quails intended for human consumption in China. In another study from the same group, Cong et al. [9] found a 9.52% infection rate in common quails from six cities in Shandong, Liaoning, and Jilin provinces in China. Alvarado-Esquivel et al. [32] reported a 14.3% infection rate in quails from Mexico. These diverse findings underscore the global prevalence of *T*. *gondii* in quail populations and highlight the importance of ongoing surveillance and research.

The analysis of our overall results revealed that *T*. *gondii* infection in common quails was not limited to a specific sampling site or bird sex (Tables 1 and 3). However, when considering the overall data for quails and data generated specifically from Multan district, a noteworthy observation emerged: wild hens exhibited a higher susceptibility to *T*. *gondii* infection compared to cocks (Table 3). This finding contradicts the results reported by Cong et al. [9] who observed variations in *T*. *gondii* infection in common quails across different sampling sites in China. Cong et al. [9] also noted that hens were more susceptible to infection than cocks.

Similarly, Lamy and Kawan [29] reported variations in *T*. *gondii* infection within different organs of quails, with the highest infection rate observed in pectoral muscles and the lowest in the heart. They also found that hens were more susceptible to infection than roosters. In another study, Lamy and Kawan [30] reported a higher *T*. *gondii* infection rate in chicks compared to mature birds, as well as a higher infection rate in birds captured in September compared to those captured in July. These contrasting findings underscore the complexity of factors influencing *T*. *gondii* infection dynamics in quail populations, including geographical location, age, and sex, necessitating further research for a better understanding of these patterns.

The complete blood count is a routine and sensitive health indicator for birds, as changes in the blood profile can be easily detected even in the absence of visible disease-associated signs and symptoms [33]. *T*. *gondii* is known for causing chronic and mostly asymptomatic infections in birds [28]. However, in the present investigation, we observed that *T*. *gondii*-infected common quails exhibited disturbances in hemoglobin, hematocrit, white blood cell count, lymphocytes (%), monocytes (%), mean cell volume, and platelet count. These effects were more pronounced among wild quails than in farmed quails, aligning with the higher prevalence of *T*. *gondii* among wild birds (Table 5).

**Table 5:** Comparison of the studied complete blood count parameters between *Toxoplasma gondii* infected and uninfected wild and farmed common quail captured from four districts in the present study.

Parameters	Positive wild samples	Negative wild samples	Positive farmed samples	Negative farmed samples
White blood cell (x 10^3^ μL^-1^)	154.5 ± 8.1	112.9 ± 3.6 **	156.4 ± 5.1	155.2 ± 2.5
Lymphocytes cells/ul	114.4 ± 8.1	89.9 ± 3.2 *	116.2 ± 3.9	117.0 ± 2.2
Monocytes	36.9 ± 9.2	11.64 ± 1.1 *	40.8 ± 5.8	40.5 ± 2.5
Granulocytes	34.1 ± 9.7	11.3 ± 2.6	38.9 ± 6.2	37.6 ± 2.4
Lymphocytes (%)	86.7 ± 9.1	78.4 ± 2.9	90.4 ± 3.3	91.1 ± 2.1
Monocytes (%)	30.7± 9.5	9.66 ± 0.78	35.2 ± 4.5	33.9 ± 2.4
Granulocytes (%)	28.9 ± 9.7	9.4 ± 2.4	33.8 ± 5.6	32.7 ± 2.4
Red blood cells (x 10^6^μL^-1^)	17.9 ± 9.3	3.089 ± 0.15	22.0 ± 2.9	21.3 ± 2.2
Haemoglobin (gdL^-1^)	28.0 ± 8.8	14.44± 0.54	31.2 ± 3.3	30.7 ± 2.2
Hematocrit	53.5 ± 8.1	45.4 ± 1.9	55.3 ± 2.2	53.6 ± 2.5
Mean cell volume (f L)	176.0 ± 9.5	153.2 ± 4.2 *	180.4 ± 4.4	186.7 ± 2.8
Mean cell haemoglobin (pg)	68.4 ± 8.7	52.8± 4.4	71.4 ± 3.7	76.6 ± 2.4
Mean corpuscular haemoglobin concentration (g/dl)	48.5 ± 8.9	33.5 ± 1.9	51.9 ± 1.7	53.4 ± 2.0
Red Cell Distribution width	23.2 ±9.7	13.30 ± 0.81	28.0 ± 2.5	27.9 ± 2.1
Red Cell Distribution width with standard deviation	67.7 ± 12	101.8± 7.2 *	77.3 ± 4.7	93.4 ± 5.6 *
Platelets Count	53.5 ± 16	146.8 ±15 **	68.8 ± 5.4	67.6 ± 3.0
Mean Platelet Volume	21.8 ± 9.5	4.53 ± 0.20	26.3 ± 6.1	25.5 ± 2.2
Platelet Distribution Width	23.8 ± 9.6	8.206 ± 0.15	28.3 ± 1.8	27.7 ± 2.2

Notes: P-value represents the output of two sample t test calculated for each studied parameter. Abbreviations

*: Least statistically significant, P < 0.05

**: Statistically significant, P < 0.01.

While the effects of this pathogen have not been previously investigated in common quails, Lashari et al. [34] reported significant disturbances in red blood cell count and hemoglobin levels in *T*. *gondii* infected ring-necked pheasants, green pheasants, and silver pheasants screened in Pakistan. The observed changes in our study clearly indicate signs of infection. The increase in white blood cell count and changes in lymphocytes and monocytes suggest the body’s response to the parasite [35]. Elevated red blood cells, hemoglobin, and mean cell volume are considered adaptations to energetic activities during parasitic invasion [36]. Changes in packed cell volume could be related to environmental factors and other factors like metabolism, workload, or genetics [37]. Elevated red cell distribution width is commonly seen as a response to infection, where pro-inflammatory cytokines inhibit erythrocyte maturation, resulting in an increase in immature erythrocytes circulating in the blood and higher red cell distribution widths [38]. Although platelets do not come into direct contact with the parasite, an increase in platelets has been observed following parasitic infections, indicating the involvement of platelets in parasitic diseases [39]. These hematological changes provide valuable insights into the impact of *T*. *gondii* infection on the health of common quails.

Genetic diversity of *T*. *gondii* isolated from a variety of vertebrate hosts has been reported and a variety of repetitive gene regions in the parasite genome including the Glycerol-3-phosphate dehydrogenase (*B1*) gene, rhoptry protein (ROPs) genes, 18S rDNA sequences and the internal transcribed spacer (*ITS-1*) [40]. During the present study, we reported the genetic diversity of *T*. *gondii* in Pakistani common quail for the first time. Phylogenetic analysis was conducted using five PCR-amplified products from the *ITS-1* gene of the pathogen. Sequences of the *ITS-1* gene have been widely used in molecular phylogenetic studies as its variability is relatively high and it can be conveniently amplified [41]. All identified haplotypes formed a cluster closely resembling the *ITS-1* gene sequences of *T*. *gondii* reported in various hosts, including cattle and goats in Pakistan (MW885249, Taalay et al. [42] and OL461229, Aziz et al. [43]), cats from China (JQ235842, *unpublished data*) and Thailand (KP895868, Chemoh et al. [44]), cats from Germany (EU025025, Schares et al. [45]), and Poland (KX459518, unpublished data).

Additionally, the identified haplotypes exhibited similarities with *ITS-1* gene sequences found in Eurasian otters from Norway (KM657806, Gjerde and Josefsen [46]), juvenile bald eagles from Canada (MN153989, *unpublished data*), California sea otters from the USA (AY488168, Miller et al. [47] and AY143140, Su et al. [48]), sea birds, dogs, cattle, milk from dairy cows, chickens, wild birds, and goats in Brazil (MH793503, MH793505, MW021420, ON809794, ON809795, MW023595, unpublished data, MF765978, Koch et al. [49], FJ966049, Santos et al. [50], JF810953, JF810956, JF810959, Gonçalves et al. [51], OL323108, Llano et al. [52], FJ176232, FJ176233, FJ176234, Silva et al. [53]), as well as in the milk of livestock from Mongolia (MH423902, unpublished data) (Fig 2). This extensive genetic diversity underscores the widespread distribution of *T*. *gondii* and its potential transmission across diverse host species and geographical locations.

*Plasmodium* spp. has been detected in both domesticated and wild birds worldwide, with Columbiformes, Galliformes, and Passeriformes reported to have the highest diversity of *Plasmodium* [35]. However, information regarding the prevalence of *Plasmodium* spp. in common quails is scarce in the literature. Therefore, this report from Pakistan marks the first time that we are presenting findings on the infection of *Plasmodium* sp. in enrolled quails, revealing a 3.5% infection rate (Table 2). The infection rate was higher in wild birds (4.2%) compared to farmed ones (2.5%), although the difference in *Plasmodium* spp. infection did not reach statistical significance (P = 0.4) (Table 2).

In the literature, only one report has addressed the presence of *Plasmodium* spp. in Pakistani birds. Sadaf et al. [7] collected and screened blood and fecal samples from doves (*Zenaida asiatica*), pigeons (*Columba livia*), ducks (*Anas platyrhynchos*), partridges (*Alectoris chukar*), turkeys (*Meleagris gallopavo*), and geese (*Chen caerulescens*), revealing a 29% infection rate with *Plasmodium juxtanucleare*. The presence of this parasite was detected among all enrolled bird species. Several Plasmodium species have been reported from birds, including poultry and farmed birds such as *Plasmodium durae*, *Plasmodium gallinaceum*, *P*. *relictum*, and *P*. *juxtanucleare* [13]. During the present study, DNA sequencing followed by BLAST analysis confirmed the presence of *P*. *relictum* in local quails.

*Plasmodium relictum* has been reported in birds in India, with Patra et al. [54] finding 20% infection in *Upupa epops*, *Pycnonotus cafer*, *Bubulcus ibis*, and *Passer domesticus* captured from the northeastern part of India. Among studies related to quails, Jubril et al. [35] reported the presence of *P*. *gallinaceum* in *Coturnix coturnix japonica* from Nigeria. Hassan et al. [31] reported 4% of quails captured from Egypt being infected with *P*. *gallinaceum*. Mohammad [55] reported a 4.45% co-infection rate with *Plasmodium* spp. in quails captured from Iraq, although the primary focus of the study was screening for the presence of microfilaria. These limited findings suggest that quails can be infected with more than one *Plasmodium* species, emphasizing the need for detailed studies from various areas, specifically in Pakistan, and globally for a better understanding of *Plasmodium* spp.-mediated infections in common quails.

The risk factor analysis for *Plasmodium* spp. infection in common quails revealed that the infection was not restricted to a specific sampling site or bird sex. Additionally, the infection was not limited to wild or farmed quails, as indicated by the findings in Tables 1 and 3. However, when conducting district-wise data analysis, it was observed that farmed quails from Layyah and wild quails from Multan exhibited higher *Plasmodium* spp. infection rates (Table 2). Notably, farmed hens from Dera Ghazi Khan were more susceptible to *Plasmodium* spp. infection than cocks (Table 4).

These results align with several previous studies that have documented a higher susceptibility of wild birds to *Plasmodium* spp. infection compared to farm ones [7, 31, 54]. However, there are contradictory reports regarding the influence of sex and age on susceptibility to *Plasmodium* spp. infection. Some studies suggest that male birds are more susceptible, while others indicate a higher susceptibility in females. Similarly, conflicting findings exist for susceptibility between young and mature birds [31, 35, 55]. This limited and divergent data underscores the need for more studies specific to birds that investigate the epidemiological factors associated with *Plasmodium* spp. infection, providing a clearer understanding of the dynamics of avian malaria in different bird populations.

The complete blood count analysis revealed that *Plasmodium* spp. infected quails exhibited elevated white blood cell counts, total lymphocytes, lymphocytes percentage, and red cell distribution width, while having reduced hemoglobin and mean cell volume compared to Plasmodium spp. uninfected birds (Table 6). These findings align with Jubril et al. [35] who reported a significant decrease in red blood cell counts, packed cell volume, and hemoglobin, along with an increase in white blood cell and eosinophil counts in birds with haemoparasites, including *Plasmodium* sp., compared to uninfected birds. Ishtiaq and Barve [56] also observed decreased hemoglobin and hematocrit levels with an increase in malarial parasite intensity in high-elevation bird populations.

**Table 6:** Comparison of the studied complete blood count parameters between *Plasmodium* spp. infected and uninfected wild and farmed common quail captured from four districts in the present study.

Parameters	Positive wild samples	Negative wild samples	Positive farmed samples	Negative farmed samples
White blood cell (x 10^3^ μL^-1^)	134.21 ± 1.9	108.7 ± 2.7 ***	131.25 ± 0.15	126.2 ± 3.9
Lymphocytes cells/ul	109.3 ± 4.2	84.2 ± 1.8 ***	104.55 ± 5.4	95.3 ± 2.8
Monocytes	17.04 ± 0.85	13.12 ± 1.0 **	16.40 ± 2.5	16.0 ± 1.7
Granulocytes	10.74± 0.91	12.3 ± 1.9	10.30 ± 2.7	16.2 ± 1.6
Lymphocytes (%)	79.27 ± 1.4	79.4 ± 1.9	79.65 ± 3.9	77.1 ± 1.7
Monocytes (%)	12.71 ± 0.65	11.52 ± 0.80	12.50 ± 1.9	10.86 ± 0.67
Granulocytes (%)	8.01 ± 0.71	10.4 ± 1.5	7.85 ± 2.1	12.05 ± 1.1
Red blood cells (x 10^6^μL^-1^)	2.357 ± 0.33	3.31 ± 0.43 *	2.34 ± 0.91	1.214 ± 0.10
Haemoglobin (gdL^-1^)	14.23 ± 1.2	16.83 ± 0.62 *	14.10 ± 3.3	11.60 ± 0.35
Hematocrit	43.3 ± 6.8	45.7 ± 1.7	43.8 ± 18	23.5 ± 1.6
Mean cell volume (f L)	181.6 ± 4.0	159.2 ± 2.6 ***	185.3 ± 7.0	190.0 ± 3.4
Mean cell haemoglobin (pg)	63.5 ± 3.8	59.7 ± 2.6	64.5 ± 11	117.61 ± 8.8 *
Mean corpuscular haemoglobin concentration (g/dl)	35.34 ± 2.7	37.57 ± 1.2	35.1 ± 7.3	61.6 ± 4.4
Red Cell Distribution width	10.10 ±2.2	9.29 ± 0.69	6.650 ±0.65	8.99 ± 0.62 *
Red Cell Distribution width with standard deviation	122.6 ± 24	93 ± 22	126.8 ± 69	179.0 ± 11
Platelets Count	30.6 ± 5.4	61.4 ± 8.9 **	23.0 ± 13	43.3 ± 3.4
Mean Platelet Volume	5.9286 ± 0.018	6.00 ± 0.46	5.95 ± 0.050	5.043 ± 0.079 ***
Platelet Distribution Width	8.357 ± 0.24	8.42 ± 0.48	8.200 ± 0.70	7.85 ± 0.22

Notes: P-value represents the output of two sample t test calculated for each studied parameter.

Abbreviations

*: Least statistically significant, P < 0.05

**: Statistically significant, P < 0.01

***: Highly statistically significant, P < 0.001.

In contrast, Muriel et al. [57] observed no significant effect of *Plasmodium* spp. infection on the body condition, hemoglobin, and hematocrit levels of Red Avadavats or common Waxbills. As various *Plasmodium* species are known to infect birds, it would be interesting to explore the specific effects of each species on the complete blood count of a particular bird species, including common quails. The variations observed in different studies may be attributed to differences in the host-parasite interactions, *Plasmodium* species involved, and the overall health and condition of the bird populations studied. Further research is warranted to comprehensively understand the impact of different *Plasmodium* species on the hematological parameters of specific bird species, such as common quails.

Phylogenetic studies of the genus *Plasmodium* have been performed using the sequences of the nuclear, mitochondrial and plastid genes as well as the adenylosuccinate lyase (*ASL*) gene that encodes an enzyme involved in the salvage of host purines needed by malaria parasites for their DNA synthesis [58]. The Cytochrome B gene is also commonly used for the phylogenetic evolution studies at intraspecific and interspecific levels as it has a moderate evolutionary level and a clear evolution pattern [59]. The sequence obtained in this study closely resembled the Cytochrome b sequences of *Plasmodium* sp. isolated from various locations, including Germany (OR416871, unpublished data), captured and free-ranging wild birds in Switzerland (OP727975, Meister et al. [60]), saurian malaria parasites from the USA (NC009961, unpublished data, KY653774, and KY653779, Pacheco et al. [61]), the UK (LN835311, *unpublished data*), Lithuania (KY653784 and KY653762, Pacheco et al. [61]), and malaria parasites from Japan (AB375765, Hayakawa et al. [62]). This limited dataset highlights the importance of collecting more common quail samples from different geoclimatic regions in Pakistan. Analyzing these samples for the genetic diversity of *Plasmodium* sp. will provide additional information for proper taxonomic identification and the development of therapeutic approaches for effective control. Expanding the genetic analysis of *Plasmodium* sp. in common quails across diverse regions will contribute to a more understanding of the pathogen’s diversity and aid in developing targeted control strategies.

Despite the valuable insights gained from our study, there are certain limitations that need to be acknowledged. Firstly, we primarily relied on the detection of *T*. *gondii* DNA in blood samples to confirm past exposure to the parasite. This approach does not provide definitive evidence of active infection or allow for the determination of prevalence rates. To accurately assess the actual prevalence of infection and understand the current infection status, serological testing, which detects specific antibodies produced in response to the infection, should be incorporated in future studies. This additional information would offer a more comprehensive understanding of the true prevalence of *T*. *gondii* infection. Secondly, we were failed to re-amplify most of the *Plasmodium* spp. isolates as in the first attempt the DNA sequencing did not went well and the resultant sequences had low query cover when they were evaluated through BLAST analysis. Lastly, our study focused on a specific population or limited geographical area, which may restrict the generalizability of our findings to other populations or regions. Future research should aim to include a more diverse range of participants and expand the study to multiple locations to enhance the external validity of the findings [63]. Despite these limitations, our study provides valuable preliminary insights into the presence of *Toxoplasma gondii* and *Plasmodium* spp. DNA in local common quails.

## 5. Conclusion

In summary, this study marks the first report of *T*. *gondii* and *P*. *relictum* infections in common quails from Pakistan, significantly contributing to our understanding of the genetic diversity, associated risk factors, and the impact of these pathogens on the host’s complete blood count profile. Both parasites were identified in both wild and farmed quails, with a higher prevalence observed in wild birds. The detection of these parasites in seemingly healthy quails underscores the potential for lifelong carriage and their role as reservoirs, posing risks for transmission through vectors or contact with other birds, particularly in communal rearing settings. To address these concerns, we advocate for expanded research encompassing common quails from diverse geographical regions in Pakistan to refine the taxonomic identification of *Toxoplasma gondii* and Plasmodium species. Such investigations are crucial for developing effective therapeutic approaches, ultimately reducing the risk of zoonotic transmission and enhancing control measures for these pathogens within the human food chain.

## Supporting information

S1 Fig The subjects used in the present investigation: Common quail. (A) a male and (B) female bird (personal clicks).
(JPG)

S1 Table Oligonucleotide primer sequences used for the detection of *Toxoplasma gondii* and *Plasmodium* spp. in the blood samples of common quails collected in the present study. Abbreviations: *ITS1*: First internal transcribed spacer of ribosomal DNA, *Cyt b*: *Cytochrome b* gene.
(DOCX)
