# Expression of Concern: Molecular prevalence, phylogeny and hematological impact of *Toxoplasma gondii* and *Plasmodium* spp. in common quails from Punjab, Pakistan

**DOI:** 10.1371/journal.pone.0354414

**Published:** 2026-07-23

**Authors:** The PLOS One Editors

The *PLOS One* Editors issue this Expression of Concern because this article [1] was identified as one of a series of submissions for which we have concerns about the peer review process. Readers are advised to interpret the article [1] with caution.
