# Supplementary figures and images for: Molecular prevalence, phylogeny and hematological impact of Toxoplasma gondii and Plasmodium spp. in common quails from Punjab, Pakistan
Source: PLoS One. 2024 May 31;19(5):e0304179. doi: 10.1371/journal.pone.0304179 (PMC11142681; doi:10.1371/journal.pone.0304179)

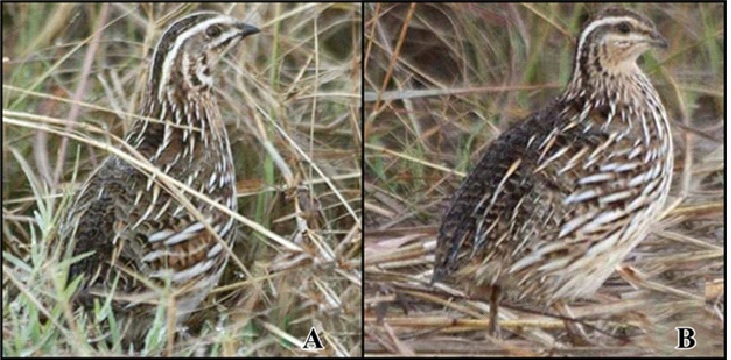

Supplement: S1 Fig — (A) a male and (B) female bird (personal clicks). (JPG) [file pone.0304179.s001.jpg]
