# Supplementary material for: Molecular prevalence, phylogeny and hematological impact of Toxoplasma gondii and Plasmodium spp. in common quails from Punjab, Pakistan
Source: PLoS One. 2024 May 31;19(5):e0304179. doi: 10.1371/journal.pone.0304179 (PMC11142681; doi:10.1371/journal.pone.0304179)
Supplement: S1 Table — Abbreviations: ITS1: First internal transcribed spacer of ribosomal DNA, Cyt b: Cytochrome b gene. (DOCX) [file pone.0304179.s002.docx]

**Supplementary Table 1.** Oligonucleotide primer sequences used for the detection of *Toxoplasma gondii* and *Plasmodium* spp. in the blood samples of common quails collected in the present study.

| **Parasite** | **Amplified marker** | **Primer sequence (5’-3’)** | **Amplicon size (bp)** | **Reference** |
| --- | --- | --- | --- | --- |
| *Toxoplasma gondii* | *ITS-1* | AGTTTAGGAAGCAATCTGAAAGCACATC  GATTTGCATTCAAGAAGCGTGATAGTAT | 300 | [21] |
| *Plasmodium* spp. | *Cyt b* | CCTCACGAGTCGATCAGG  GGAAACCGGCGCTAC | 377–379 | [22] |

Abbreviations: *ITS1*: First internal transcribed spacer of ribosomal DNA, *Cyt b*: *Cytochrome b* gene
